# The relationship between role ambiguity, emotional exhaustion and work alienation among chinese nurses two years after COVID-19 pandemic: a cross-sectional study

**DOI:** 10.1186/s12888-023-04923-5

**Published:** 2023-07-18

**Authors:** Hong-li Zhang, Chao Wu, Jia-ran Yan, Jun-hua Liu, Pei Wang, Meng-yi Hu, Fang Liu, Huan-min Qu, Hong-juan Lang

**Affiliations:** 1 School of Nursing, Shaanxi University of Chinese Medicine, Xianyang, China; 2 Department of Nursing, Fourth Military Medical University, No.169 Changle West Road, Xi’an, 710032 Shaanxi China; 3 Department of Nursing, Qufu Traditional Chinese Medicine Hospital, Shandong, China; 4 Human Resources Department, Tangdu Hospital, Air Force Medical University, Shaanxi, 710032 Xian China

**Keywords:** Nurse, Role ambiguity, Emotional exhaustion, Work alienation

## Abstract

**Background:**

work alienation is receiving increasing attention as a psychological risk at work, and little is known about the mechanisms of role ambiguity and work alienation in nurses in the context of the COVID-19 pandemic. This article aims to examine how role ambiguity affects work alienation among Chinese nurses during the two years after COVID-19 pandemic and verify emotional exhaustion as mediators.

**Methods:**

A cross-sectional study design was used to recruit 281 Chinese nurses. Nurses completed online questionnaires containing demographic characteristics, role ambiguity, emotional exhaustion, and work alienation, and SPSS 26.0 and AMOS 24.0 were used for data analysis and structural equation modelling.

**Results:**

work alienation scores were (34.64 ± 10.09), work alienation was correlated with role ambiguity and emotional exhaustion (*r*1 = 0.521, *r*2 = 0.755; *p* < .01), and role ambiguity was positively correlated with emotional exhaustion (*r* = 0.512; *p* < .01). A mediating effect of emotional exhaustion between role ambiguity and work alienation held (mediating effect of 0.288, 95% CI: 0.221–0.369, accounting for 74.8% of the total effect).

**Conclusion:**

Role ambiguity has a significant direct effect on nurses’ feelings of alienation and exacerbates alienation through emotional exhaustion. Clarifying roles at work and being less emotionally drained are effective ways to reduce nurses’ feelings of alienation.

## Introduction

As the population grows and ages, the demand for health care services rises, promoting the development of the nursing profession while placing increasing demands on nurses [1]. However, the shortage of nurses is a key challenge for global health systems [2]. The World Health Organization reported 9 million nurses shortage by 2030 and the severely understaffed situation in nursing profession, which was made worse by the advent of COVID-19 [3]. People have become more connected as a result of advancements in transportation and lifestyle changes, and in the future, the appearance of novel infectious diseases won’t be as surprising [4]. During the pandemic, nurses played a pivotal role in the prevention, treatment and care of infected patients, creating a safer health care organization [5]. The literature points to increased risk of skin damage from wearing uncomfortable protective gear for long periods of time in a closed environment, increased workload and reversed sleep rhythms, leading to physical and mental fatigue, periodic emotional outbursts and increased fear among nurses [6]. They are very careful in their work to prevent themselves from becoming infected and in turn infecting their family and friends [7]. In addition, they suffer discrimination and prejudice from the public because of their close contact with infected patients, but their sense of mission and responsibility as health care professionals makes them conflicted and tormented inside [8]. The imbalance between what they give and what they get in return, the difficulty in obtaining satisfaction from their work and the sense of disparity in expectations, reinforce the desire to leave the nursing profession and a sense of work alienation emerges [9].

Work alienation is a negative state in which one’s needs and expectations are not met at work, resulting in reduced behavioural engagement and psychological alienation [10, 11]. There are three core dimensions of work alienation: (a) sense of helplessness, which refers to the fact that individuals do not have control and control over the content of their work and that they do not have the right to make decisions about it; (b) sense of friendlessness, where the individual do not consider themselves a member of the organization, thus having no sense of identity or not appreciating the ideas, goals and values of the organization; (c) sense of meaninglessness means that individuals do not have a plan to achieve their personal career goals, are not motivated to achieve organizational goals, do not appreciate the importance of their work, and do not feel the value of their work [12]. This concept has been explored extensively over the last 10 years and in Turkey over 80% of nurses feel alienated at work [12]. In China, nurses reported a medium to high level of alienation [13]. In the Netherlands, alienation midwives were less engaged in their work [10]. Studies in Spain, Italy and Pakistan showed high rates of work alienation [14–16]. Research also noted that nurses are prone to experience work alienation because of work overload and occupational risks [17]. More importantly, the study pointed out that employees performed poorly at work or even showed anti-productive behaviour when they had stronger feelings of alienation [18]. Therefore, in the context of major public health emergencies, it is necessary to investigate the potential influencing factors of nurses’ work alienation, and strategies need to be tapped to effectively alleviate nurses’ alienation, which would subsequently protect patients safety, reduce nurse turnover and promote the development of nursing.

Role ambiguity is a lack of clarity related to the absence of job information such as role responsibilities or expectations [19]. The current changes in the health care system have expanded and complicated the scope of nurses’ roles. As a result, nurses found themselves doing overlapping tasks, receiving missing and receiving confusing treatment information (e.g. COVID-19 patient treatment and care information, roles in multidisciplinary teams, unclear instructions from managers) [20–22]. Previous research has found that role ambiguity has a negative impact on an individual’s physical and psychological well-being and is negatively associated with work attitudes and behaviours (such as work alienation, organizational commitment), while being positively associated with job dissatisfaction [23–26]. Lacking clear information about the job match, role ambiguity makes it difficult for individuals to perform their jobs, lose confidence in their ability to do the job, become frustrated and feel incompetent due to their inability to control the job, and gradually show alienation from the work and develop feelings of work alienation [27]. Previous research has explored the relationship between role ambiguity and work alienation, but this relationship is not yet clear in the nurse population.

Emotional exhaustion is the depletion of psychological and emotional resources caused by the demands of work and is one of the core factors of burnout [28]. Numerous studies have found that emotional exhaustion has a negative impact on an individual’s physical and mental health and reduces employee motivation and engagement, psychological withdrawal, avoidance of work and ultimately leads to a sense of alienation from work [29, 30]. Marx argues that workers experience alienation followed by physical exhaustion and a lack of mental strength [31]. Based on this, some studies have found that emotional exhaustion increases the occurrence of work alienation, and that work alienation also exacerbates emotional exhaustion in employees [14]. According to the Theory of Limited Self-Control Resource, emotional exhaustion occurs when individuals are under the influence of stressors (role ambiguity) and need to continuously consume psychological resources to make adjustments to their behaviour and cognition in order to exhibit rational work behaviour, with a long-term depletion of intrinsic resources [32]. According to Chiara Panari’s research [33], role ambiguity decreases the motivation of health care workers, and health care workers feel exhausted and depressed in response to role ambiguity, leading to emotional exhaustion. Anita Padmanabhanunni’s study of university teachers during the COVID-19 epidemic found that role ambiguity increased teacher stress, impairs mental health, and was a significant predictor of teacher emotional exhaustion [34].

As role ambiguity increases emotional exhaustion, which is negatively associated with work alienation, emotional exhaustion may mediate the relationship between role ambiguity and work alienation. However, to date, to our knowledge, this mechanism has been under-researched in the nurse population, both in China and in other countries.

### Study aims

**Fig. 1:**
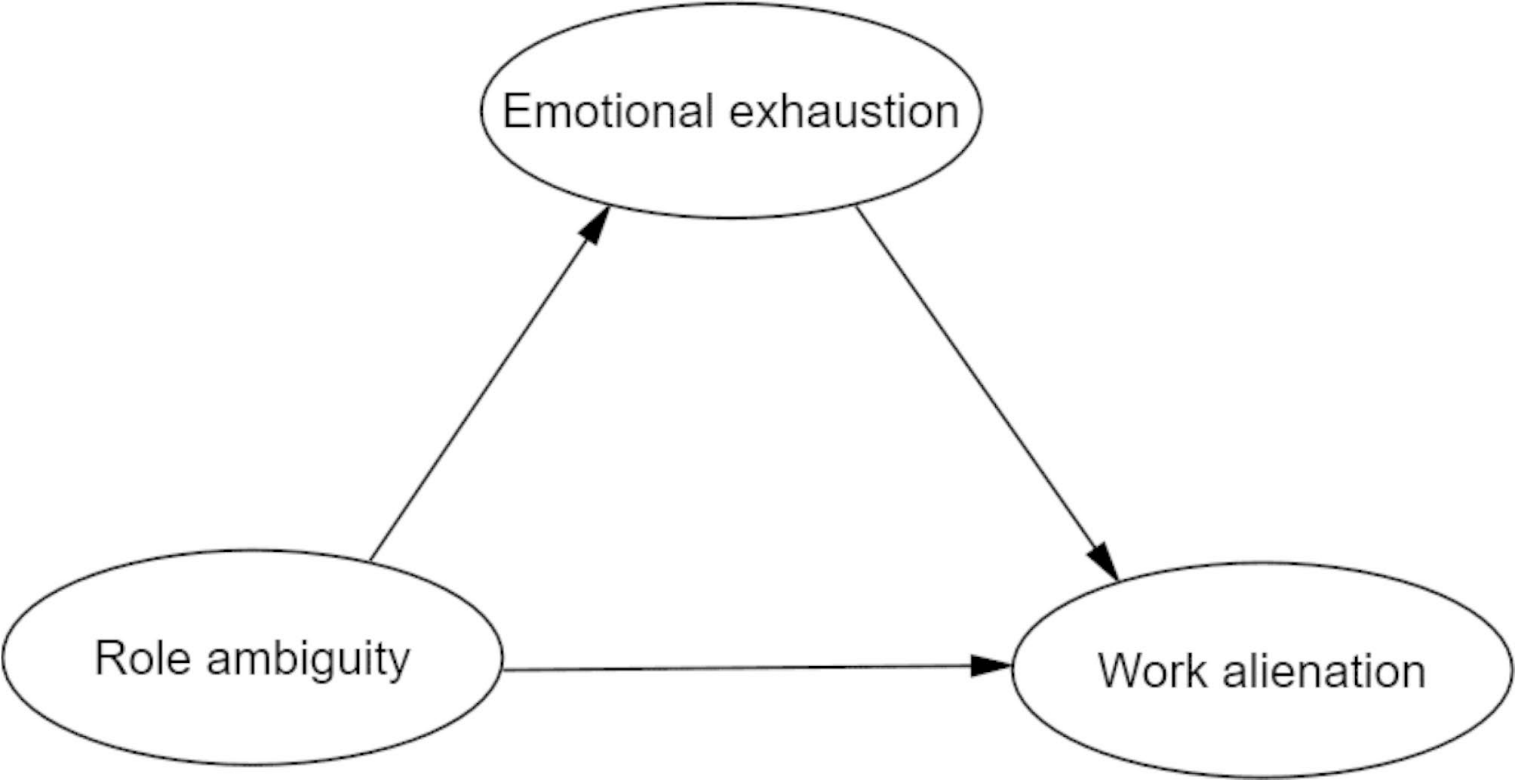
The hypothesized model

The study is designed (1) to identify the current state of role ambiguity, emotional exhaustion and work alienation among nurses; (2) to investigate the mechanisms by which role ambiguity affects job alienation among nurses and to validate the mediating role of emotional exhaustion. The theoretical model is shown in Fig. 1.

## Methods

### Design and sampling

The study was a cross-sectional design, using a questionnaire to collect data. A convenience sampling method was used to select four grade 3 A hospitals in Shandong Province and Shaanxi Province from October 2022 to January 2023. With the help and support of hospital administrators, a link to an anonymous version of the electronic questionnaire was sent to clinical nurses. Inclusion criteria: (1) nurses who had obtained the professional qualification certificate of the People’s Republic of China; (2) nurses who had been working in the hospital for more than 1 year; (3) informed consent and voluntary participation. Exclusion criteria: (1) those who were not in their posts during the survey period (due to sick leave, maternity leave, etc.); (2) rotating nurses or trainee nurses.

We have set each IP address to be filled in only once in the backend of the questionnaire software in order to prevent receiving duplicate questionnaires. In addition, intelligent logic recognition was set in the backend to check for invalid (all questions answered the same, does not correspond to reality, “Age 2000”, etc.) questionnaires. Finally, valid questionnaires were checked by two researchers to ensure the accuracy of the data.

### Sample size

We calculated the sample size according to Kendall (1975) [35]. The total number of questionnaire entries used in this study was 27, which was then expanded by a further 20% given the potential loss of sample size due to incomplete completion.

The sample size was therefore calculated as N = [27 × 5 × (1 + 20%)] = 162, indicating that a minimum of 162 participants were required for this study. According to the Muelle [36], the proposed structural equation model (SEM) requests a sample size of no less than 200 for the framework, and, ultimately a total of 281 nurses met the inclusion and exclusion criteria to take part in our study.

### Measurements

#### Covariables: social-­demographic characteristics

Sociodemographic variables include mainly gender, age, degree (college, bachelor, master and above), marital status (married, unmarried), job title, years of work (0 ~ 2,3 ~ 5,6 ~ 10, > 10).

#### Dependent variable: work alienation

Work alienation was measured using the Work Alienation Questionnaire for Nurses developed by Ren [37], which is widely used in China and has good reliability and validity [38]. The questionnaire consisted of 12 items from 3 dimensions: feelings of helplessness (4 items), feelings of hopelessness (4 items) and feelings of meaninglessness (4 items). Scoring was based on a 5-point Likert scale from 1 (not conforming) to 5 (very conforming), with total scores of 12–27, 28–43 and 44–60 representing low, medium and high levels of work alienation among nurses respectively. The Cronbach’s alpha for the scale was.841.

#### Independent variable: role ambiguity

Role ambiguity was measured using five entries from the Role Stress Scale developed by Pteterson [39]. (for example, “I know what my job responsibilities are”, “I know exactly what the organization expects of me”), the scale has been translated into a Chinese version and is widely used by scholars in China [27]. Each entry was scored from 1 (strongly disagree) to 5 (strongly agree) and all entries were reversely coded to calculate a total questionnaire score, with higher scores associated with higher levels of role ambiguity. The Cronbach’s alpha for the scale was 0.932.

#### Mediator: emotional exhaustion

Emotional exhaustion was measured using the Maslach scale [40]. We used five items from the Maslach Burnout Inventory (MBI) (for example, “Work makes me feel physically and mentally exhausted”, “The thought of facing the day ahead makes me very tired”), it has high reliability and validity and is widely used in China [41]. The scale is scored on a five-point Likert scale from 1 (never) to 5 (always), with higher total scores indicating higher levels of emotional exhaustion. The scale’s Cronbach’s alpha for the scale was 0.957.

### Ethical approval

This study was conducted in accordance with the ethical standards required in the Declaration of Helsinki. This study is not related to human clinical trials or animal experiments. All participants were provided with anonymous written informed consent and informed that they could refuse or withdraw from the study at any time before the study was conducted, and this study was approved by the Independent Ethics Committee of the Second Affiliated Hospital of the Fourth Military Medical University.

### Data analysis

For the data analysis, IBM SPSS Statistics 26.0 was employed. Initially, we examined demographic characteristics by descriptive statistics and analyzed the correlation between role ambiguity, emotional exhaustion, and work alienation by Pearson correlations. Finally, we used IBM AMOS 24.0 to build and measure structural equation models with 2000 bootstrapping resamples to calculate the mediating effect of emotional exhaustion, with 95% CI not including 0, representing a significant mediating effect.

## Results

### Sociodemographic characteristics

A total of 281 nurses participated in this study, of whom 243 (86.5%) were female, with a mean age of 32.88 years (SD = 6.93), a mean length of service of 10.15 years (SD = 7.63), and 204 (72.6%) were married, and most nurses had obtained a bachelor’s degree199 (70.8%), as shown in Table 1.

**Table 1:** Sociodemographic characteristics of the participants

Variable	Category	N	%
Gender	Male	38	13.5
	Female	243	86.5
Age (years)	20 ~ 30	107	38.1
	31 ~ 40	138	49.1
	≥ 41	36	12.8
Educational levels	Junior college	68	24.2
	Undergraduate	199	70.8
	Master degree or above	14	5.0
Marital status	Unmarried	77	27.4
	Married	204	72.6
Working age (years)	≤ 2	50	17.8
	3 ~ 5	36	12.8
	6 ~ 10	86	30.6
	≥ 11	109	38.8

### Means, standard deviations, and correlations among variables

As shown in Table 2, the total scores for role ambiguity, emotional exhaustion, and work alienation were 12.22 ± 4.74, 16.37 ± 4.75, and 34.64 ± 10.09, respectively. Correlation analyses revealed positive correlations between role ambiguity, emotional exhaustion and work alienation (*r* = .521, 0.755, *p* < .01) and positive correlations between role ambiguity and emotional exhaustion (*r* = .512, *p* < .01).

**Table 2:** Correlations among study variables

Variables	Mean (SD)	1	2	3	4	5
1.Role ambiguity	12.22 (4.74)					
2.Work alienation	34.64 (10.09)	0.521**				
3. Sense of helplessness	14.10 (3.37)	0.440**	0.810**			
4. Sense of friendlessness	10.29 (3.89)	0.495**	0.900**	0.588**		
5. Sense of meaninglessness	10.25 (4.23)	0.437**	0.910**	0.593**	0.758**	
6.Emotional exhaustion	16.37 (4.75)	0.512**	0.755**	0.740**	0.591**	0.667**

**p<0.01

### Model test

**Fig. 2:**
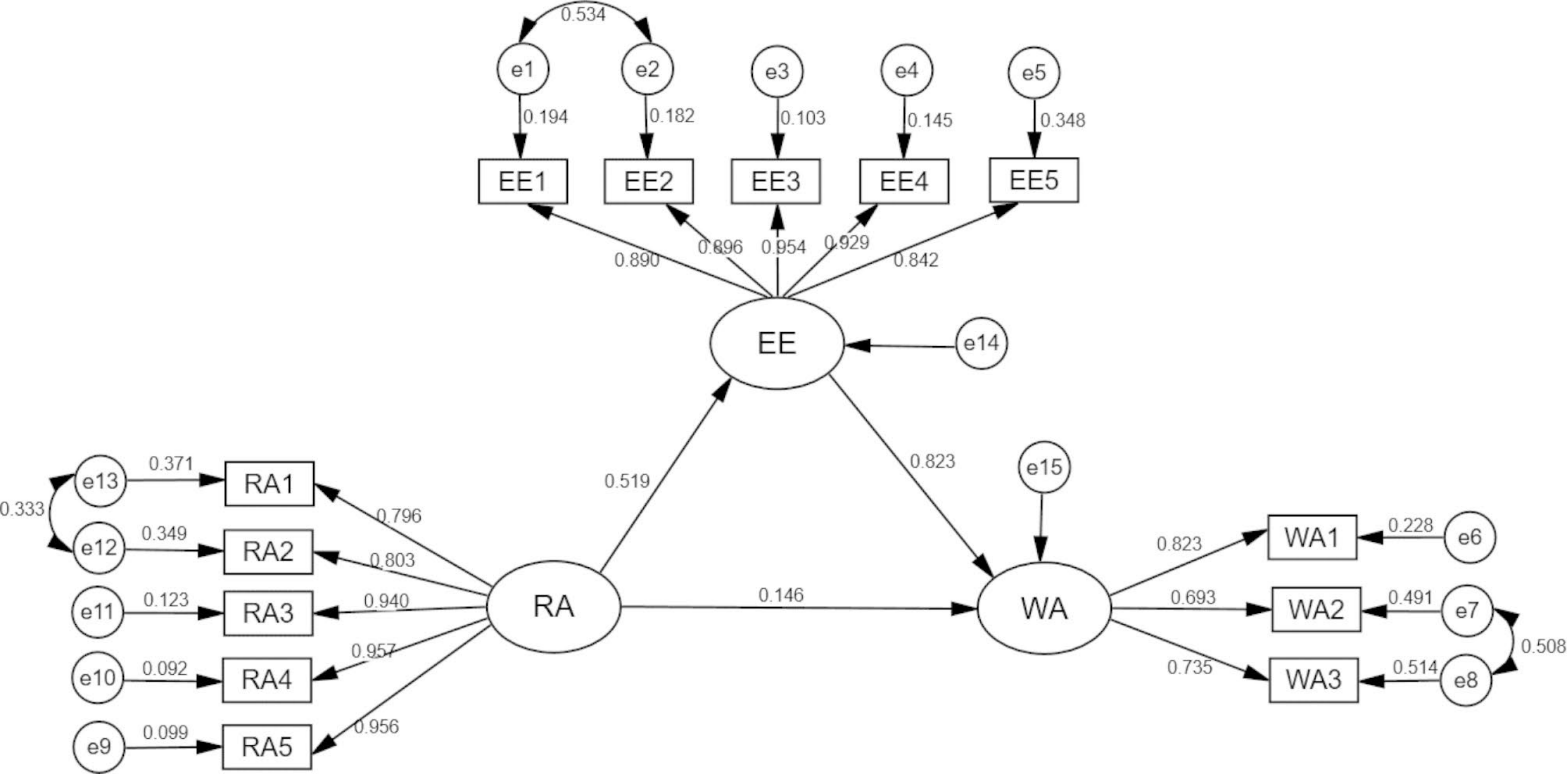
Mediation Model. RA, role ambiguity; e9-e13, manifest variables of role ambiguity; EE, emotional exhaustion; e1-e5, manifest variables of emotional exhaustion; WA, work alienation; e6-e8, manifest variables of the three dimensions of work alienation

Independent variable role ambiguity, the dependent variable work alienation, and the intermediate variable emotional exhaustion were included in the equation by AMOS 24.0 to model the structural equations. We then used maximum likelihood to fit and correct the initial model. The model fit results were: χ^2^/df = 2.155, CFI = 0.983, TLI = 0.978, SRMR = 0.040, RMSEA = 0.064 (Table 3). As shown in Table 4, the mediation analysis revealed a significant total effect of role ambiguity on work alienation (*β* = 0.385, *p* < .01). In addition, role ambiguity was directly and positively related to work alienation (*β* = 0.098, *p* < .01), positively related to emotional exhaustion (*β* = 0.432, *p* < .01), and emotional exhaustion was positively related to work alienation (*β* = 0.666, *p* < .01). The mediating effect of emotional exhaustion in role ambiguity and work alienation (2 indirect paths multiplied) was 0.288 with 95% CI not including 0 (0.221 to 0.369), indicating a significant indirect effect of role ambiguity on work alienation through emotional exhaustion, with the mediating effect accounting for 74.8% of the total effect. Our findings explain the hypothesis. Figure 2 depicts the mediating effect model.

**Table 3:** Fitting index of the models

Index	*χ* ^*2*^ */df*	CFI	TLI	SRMR	RMSEA
The Measurement model	2.155	0.983	0.978	0.040	0.064
Fitting criteria	<5.0	>0.80	>0.80	<0.08	<0.08

**Table 4:** Mediation analysis of emotional exhaustion on the relationship between role ambiguity and work alienation (n = 281)

				95%CI		
Effect	Model path	*β*	*SE*	LLCI	ULCI	*p*
Direct effect	Role ambiguity-Work alienation	0.098	0.036	0.031	0.172	<0.01
Indirect effect	Role ambiguity-Emotional exhaustion	0.432	0.046	0.346	0.523	<0.01
	Emotional exhaustion-Work alienation	0.666	0.063	0.543	0.788	<0.01
Total effect	Role ambiguity-Work alienation	0.385	0.042	0.312	0.475	<0.01

## Discussion

### Levels of role ambiguity, emotional exhaustion, and work alienation

Our research results demonstrate that Chinese clinical nurses had a moderate level of work alienation, with an overall score of 34.64 ± 10.09, similar to Xia’s findings [17], But higher than Cui’s study of nurses in northwest China [42]. We believe that a number of reasons account for this: (1) Although all were in the context of a pandemic, differences in the timing of the survey, the region studied, the intensity of the epidemic, the strains of infection, and the preventive and control measures taken may have led to differences in the number of infections, psychological stress and nurses’ workload, resulting in heterogeneity in the nurses’ sense of alienation from their work. During the period of our study, China implements the " Category B, B Control” programme (Isolation measures are not being implemented for COVID-19 patients), and the number of new coronary pneumonia cases was on the rise, with many nurses reporting COVID positive. Nurses face a dramatic increase in workload, the risk of their own infections, and work with illness, making a huge contribution and dedication to safeguarding people’s lives. However, low pay and benefits, few opportunities for advancement and limited career development make nurses experience a reduced sense of professional value, which may lead to nurses seeing their work as meaningless [38]; (2) Nurses had limited control and autonomy over their work, which constrained their enthusiasm for their work and they became less-motivated to achieve professional success, as they worked under the direction of medical advice, constantly repeated the implementation of medical advice and were responsible for checking for omissions. a sense of helplessness at not being able to choose what to do with your work [10]; (3) The lack of humanistic care for nurses in the organization, the lack of improvement in the psychosocial working environment for nurses. Particularly during the COVID-19 epidemic, the needs of patients and families increase and nurses are not supported and cared for while performing overloads, demands for quality care, and potential infection risks. Their needs are not taken into account and they are constantly exposed to psychosocial risks, leaving nurses physically and mentally exhausted and with a sense of friendlessness [38]. This may also be the reason for the prolonged work alienation among the group of nurses.

Our study reported nurses’ role ambiguity at a moderate level, higher than that in the Jing’s findings which consisted of 227 nurses from two public hospitals in northern China [43], but lower than the results of Saad A. Alyahya’s study of nurses in primary health care centres in Saudi Arabia [21]. Our findings suggest that nurses are affected by role ambiguity psychological stressors at work, which we speculate may be related to the following: (1) There are differences in guidelines and management protocols related to the treatment and care of COVID-19 patients, which are constantly and dynamically adjusted to the actual situation and, for nurses, require adaptation to organizational changes. These differences and organizational changes may lead to a lack of clarity in the delineation of job-related content, responsibilities, etc., leading to the occurrence of nurse role ambiguity [44]; (2) Today there is a high and diverse demand for healthcare. As medical professionals, nurses undertake multiple roles such as educators, caregivers, consultants, change agents, managers [45, 46]. Nurses need to use knowledge from different disciplines to perform these roles and when there is a lack of relevant knowledge, it leads to nurses being ambiguity about the role [45].

In our study, nurses’ emotional exhaustion was at a moderately high level, slightly higher than in Yang’s findings [47]. It may be caused by the following: (1) The 72.6% of married nurses in this study, during the COVID-19 epidemic, had high job demands, worked long hours in a cordoned off environment, worked endless shifts and had difficulty balancing work-family and may have been highly emotionally internalized, leading to emotional exhaustion [48]; (2) Inadequate staffing, cancellation of breaks or coming to work even when sick, unsafe working environment, lack of support from work resources, resulted in the nurses’ frustration and disillusion with their work [49]; (3) Related research points out that COVID-19-related fear, panic, misinformation and misdirected anger can all lead to workplace violence, and that workplace violence damages nurses’ mental health with negative emotions such as anxiety and depression, increasing ego depletion and causing nurses to experience emotional exhaustion [50, 51] .

### The correlations of role ambiguity, emotional exhaustion, and work alienation

The results of the correlation study showed that nurses’ work alienation and its three dimensions were positively correlated with role ambiguity, which is consistent with previous research findings [52]. Lack of clarity in job-related knowledge, responsibilities, instructions and information can prevent nurses from performing their clinical work correctly. Especially during a pandemic, when the increased scope of roles, differences in treatment policies, inadequate protective equipment support, as well as fear and dread, lack of information and lack of clarity about the job lead to nurses feeling difficult to control their work, feeling that it is meaningless and creating a sense of helplessness and friendlessness that they have no way to cope with the problem feelings. This leads to an acceleration of their alienation from their work [27]. This suggests that managers should rationalize tasks and time and make timely adjustments to changes in the work environment to reduce role ambiguity.

Work alienation was positively correlated with emotional exhaustion, meaning that nurses’ emotional exhaustion increased with the severity of work alienation, which is consistent with previous research findings [29]. The study noted that emotional exhaustion negatively affects an individual’s cognition, thinking, attention, work planning and ability to switch tasks and solve problems, depleting the individual’s internal resources, reducing work ethic and creating feelings of alienation [53, 54]. According to Marx’s theory [55], alienated employees manifest themselves as exhausted and mentally drained. During the COVID-19 epidemic, health care workers were involved in epidemic prevention, but also in completing their own work and demonstrating professionalism in their ongoing interactions with patients and families, this led to health care workers being physically overworked and suppressing themselves [56]. According to resource conservation theory, when resources are depleted, nurses are more likely to choose to reduce their commitment to patients to protect themselves from distress, become apathetic to their work and gradually alienation from it [38].

Role ambiguity was positively correlated with, indicating that increased role ambiguity was associated with higher emotional exhaustion [57]. Role ambiguity is a psychosocial risk factor for well-being [58]. Research has found that role ambiguity creates uncertainty and doubt about job goals, and how to accomplish tasks, and that employees exert effort to cope with role stress, experiences that increase occupational stress and manifest as physical exhaustion and emotional draining [59]. The need for nurses to constantly adapt to an uncertain environment, role ambiguity and lack of clear goals deplete nurses’ psychological resources [60]. During the pandemic, high role ambiguity and low personal resources had a greater impact on the mental health of nurses, who were more likely to be exhausted, emotionally disturbed, with inadequate resource support, experiencing emotional exhaustion [33].

### The mediating role of emotional exhaustion between role ambiguity and work alienation

The results of the mediating effects we analyzed showed that role ambiguity was a positive direct predictor of feelings of work alienation (0.098) and that emotional exhaustion partially mediated the relationship between role ambiguity and work alienation (0.288). Specifically, higher role ambiguity was associated with greater severity of emotional exhaustion, thereby increasing the occurrence and development of work alienation in nurses. Clear job roles help individuals to engage in their work and enhance their intellectual and emotional energy, whereas role ambiguity prevents individuals from choosing coping resources and strategies to solve problems and leads to burnout and negative emotions, reduced motivation and feelings of work alienation [27]. During a pandemic, nurses face uncertainty at work (e.g. treatment options, unclear instructions) which increases job insecurity, prevents nurses from adopting protective measures and strategies for problem solving, makes it difficult to perform clinical tasks correctly and effectively, creates stress and emotional distress from feeling unable to perform and complete their work, depletes nurses’ physical and mental resources, increases job dissatisfaction and leads to emotional exhaustion [33, 58]. These problems lead to feelings of helplessness and friendlessness, negative job evaluations, a sense of the meaninglessness of work and increase the level of work alienation among nurses.

## Conclusion

This study examines the impact of role ambiguity on nurses’ work alienation two years after the COVID-19 epidemic, and the mechanisms mediating emotional exhaustion. It fills a gap in current research and has significant reference value for improving the psychosocial working environment of nurses during major public health emergency events. Our findings indicate that nurses have moderate levels of role ambiguity and work alienation, have moderately high levels of emotional exhaustion, and that measures should be taken to improve this situation. In addition, we found that role ambiguity directly or indirectly affects nurses’ sense of work alienation through emotional exhaustion. Therefore, by reducing role ambiguity at work and improving emotional exhaustion, nurses’ sense of work alienation can be reduced, this helps to increase nurses’ enthusiasm and commitment to their work, which in turn improves the quality of care.

## Limitation

There are several limitations to this study. First, we used a cross-sectional research design and did not infer a causal relationship between the variables. Secondly, data were distributed and collected using a questionnaire platform, and the number of questionnaires distributed and the differences between nurses who participated and those who declined to participate in the study are unclear. In addition, the results may be somewhat subjective based on the self-reported questionnaire format, and these factors may limit the generalisability of the findings. Finally, our study was conducted in only two provinces in China and the findings may not be applicable to other regions and countries and have limitations. In future studies, we will expand the scope of the survey, increase the sample size, include more influencing factors and enrich the research mechanism of work alienation.

## Data Availability

The datasets used and/or analyzed during the current study are available from the corresponding author on reasonable request.
